# Construction of a simulation model and evaluation of the effect of potential interventions on the incidence of diabetes and initiation of dialysis due to diabetic nephropathy in Japan

**DOI:** 10.1186/s12913-017-2784-0

**Published:** 2017-12-16

**Authors:** Takehiro Sugiyama, Sayuri Goryoda, Kaori Inoue, Noriko Sugiyama-Ihana, Nobuo Nishi

**Affiliations:** 1 Diabetes and Metabolism Information Center, Research Institute, National Center for Global Health and Medicine, 1-21-1 Toyama, Shinjuku-ku, Tokyo, 162-8655 Japan; 2 Department of Public Health/Health Policy, Graduate School of Medicine, the University of Tokyo, 7-3-1 Hongo, Bunkyo-ku, Tokyo, 113-0033 Japan; 3 Center for International Collaboration and Partnership, National Institute of Health and Nutrition, National Institutes of Biomedical Innovation, Health and Nutrition, 1-23-1 Toyama, Shinjuku-ku, Tokyo, 162-8636 Japan; 4 Department of Bio-informational Pharmacology, Medical Research Institute, Tokyo Medical and Dental University, 1-5-45 Yushima, Bunkyo-ku, Tokyo, 113-8510 Japan; 5 Division of Endocrinology and Metabolism, Tama-Hokubu Medical Center, Tokyo Metropolitan Health and Medical Treatment Corporation, 1-7-1 Aoba-cho, Higashimurayama City, Tokyo, 189-8511 Japan; 6 Department of Diabetes, Endocrinology, and Metabolism, Center Hospital, National Center for Global Health and Medicine, 1-21-1 Toyama, Shinjuku-ku, Tokyo, 162-8655 Japan

**Keywords:** Diabetes mellitus, Diabetic nephropathies, Dialysis, Systems analysis

## Abstract

**Background:**

The prevalence of diabetes mellitus is a growing public health concern in Japan. We developed a simulation model to predict the number of people with diabetes and those on dialysis due to diabetic nephropathy. In addition, we used the model to simulate the impact of possible interventions on the number of people with diabetes and those on dialysis due to diabetic nephropathy in the near future.

**Methods:**

A simulation model with aging chains for diabetes management was built using system dynamics. The model was calibrated to population data from 2000 to 2015 (sex- and age category-specific population, the prevalence of diabetes, and the number of patients on dialysis due to diabetic nephropathy). We extrapolated the model up to 2035 in order to predict future prevalence of diabetes and related dialysis (base run). We also ran the model, hypothesizing that incidence of diabetes and/or related dialysis would be reduced by half from 2015 to 2025 and that this rate would be maintained until 2035, in order to investigate the effects of hypothetical interventions on future prevalence.

**Results:**

The developed model forecasted the population with diabetes to increase until 2028 (5.58 million males and 3.34 million females), and the population on dialysis due to diabetic nephropathy to increase until 2035 (113,000 males and 48,000 females). Simulation experiments suggested that diabetes prevention interventions would decrease the number of patients on dialysis in 2035 by 13.8% in males and 12.6% in females compared to the base run. In contrast, interventions aiming to avoid dialysis initiation for patients with diabetes would decrease the number of patients on dialysis by 37.8% in males and 38.1% in females.

**Conclusions:**

We successfully developed a simulation model to project the number of patients with diabetes and those on dialysis due to diabetic nephropathy. Simulation experiments using the model suggested that, as far as the perspective of the next 20 years, intervention to prevent dialysis is an important means of bending the increasing curve of dialysis in the population with diabetes. Simulation analysis may be useful when making and evaluating health policies related to diabetes and other chronic diseases.

**Electronic supplementary material:**

The online version of this article (10.1186/s12913-017-2784-0) contains supplementary material, which is available to authorized users.

## Background

Prevalence of diabetes mellitus is a growing global public health concern, especially in terms of population health and health economics. The International Diabetes Federation (IDF) reported that 415 million adults were living with diabetes worldwide in 2015, and IDF forecasts that this number will rise to 642 million in the next 25 years [1]. Diabetes causes serious complications such as microangiopathy (retinopathy, nephropathy, and neuropathy), macroangiopathy (coronary heart disease, stroke, and peripheral arterial diseases), and infectious diseases. These complications lead to decreased quality of life and early death for patients, as well as increased economic burden to society.

According to the National Health and Nutrition Survey, 16.3% of males and 9.3% of females in Japan were suspected to have diabetes in 2016, summing to 10.0 million Japanese adults [2]. This number is predicted to increase in spite of the population decline in Japan, which began around 2005. In addition, dialysis is exceptionally prevalent in Japan; over 320,000 individuals were on hemodialysis or peritoneal dialysis in 2015 [3]. In contrast, the frequency of kidney transplants has decreased since 2006, with less than 150 performed in 2015 [4]. Diabetic nephropathy represents a leading cause of ongoing dialysis, accounting for 38% of dialysis usage in Japan in 2015 [3]. Since health insurance and other governmental benefit programs reimburse patients on dialysis in Japan, economic burden does not likely represent a barrier to accessing dialysis as long as health insurance is sustained. Patients on hemodialysis must spend 3 to 4 h in the clinic, 3 times per week; it is therefore important to decrease dialysis usage to maintain patients’ quality of life and slow the growth of medical expenditures.

System dynamics modeling is suitable for addressing dynamically complex problems and finding an effective target for intervention [5, 6]. Using a system dynamics model related to the diabetes epidemic, Jones et al. compared the effects of interventions (obesity prevention, increased management of prediabetes, and enhanced clinical management of diabetes) on the prevalence of diabetes, which found that obesity prevention may be the most effective intervention of the three [7]. In Japan, however, there have been few studies comparing the policy choices related to diabetes management based on simulation models. Therefore, we developed a simulation model of diabetes management focusing on dialysis initiation in patients with diabetes in Japan. In addition, we compared the impact of possible interventions on the number of dialysis patients due to diabetic nephropathy in the near future.

## Methods

This was a simulation experiment using a system dynamics model that calibrated to recent statistics in Japan and their derivatives. The model used data from Japanese citizens, in order to make projections from 2000 to 2035. The scope of this model is Japanese population aged 20 years or older. The simulation model considered both type 1 diabetes and type 2 diabetes. We have two reasons for this; first, statistics from the government are not disaggregated by type of diabetes; second, the proportion of type 1 diabetes in Japan is estimated to be less than 5% [8], which implies that the heterogeneity pertaining to the type of diabetes is not likely to influence the validity and usefulness of the model greatly. All simulations were performed using Vensim® DSS for Macintosh Version 6.2 (Ventana Systems, Inc. Harvard, MA).

### System dynamics model

System dynamics is a method of depicting diagrams and constructing policy-oriented computer simulation models, using nonlinear, multiple simultaneous ordinary differential equations [6, 9]. One of the characteristics of the method is the stock-and-flow structure of the diagram; a variable enclosed in a rectangle (called the “stock” variable) represents the amount or population of the variable being studied at a certain point in time. The amount change is regulated by a wide arrow (called “flow”) that goes into (“inflow”) or out of (“outflow”) the stock variable. The flow variable represents the increment or decrement of the stock variable in the unit time. The dimension of the flow variable is always that of the stock variable differentiated by the time variable. A thin arrow from variable A toward variable B indicates that variable B is a function of variable A. If there is no arrow going into a variable, the variable is “exogenous” (i.e., a constant or a list of values). If an exogenous variable C is a list of values, it explains another variable D in concert with another variable E (the time variable in most cases), that is, the value of variable D is determined by the value of variable E from the values listed in variable C.

System dynamics models are often used to integrate the concept of positive feedback (representing virtuous or vicious cycles; the “reinforcing loop”) or the concept of negative feedback (the “balancing loop”). The model is also used to demonstrate the maturing of a population; this type of system dynamics model is called an “aging chain” [5]. In this study, we used an aging-chain system dynamics model to represent the dynamics of the Japanese population with regard to patients with diabetes and patients on dialysis due to diabetic nephropathy.

### Stock-and-flow structure

**Fig. 1:**
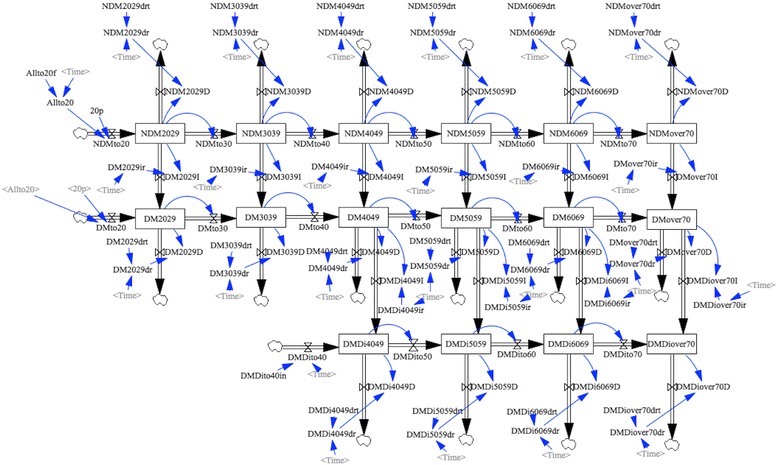
Stock-and-Flow structure of the system dynamics model

Figure 1 illustrates the stock-and-flow structure of the system dynamics model used in this study. As described above, the model is made of chains of population stocks representing stages stratified by health conditions and age categories, as well as flows describing the movement of people into and out of these stages. Age was categorized into 6 groups: 20–29 years (2029), 30–39 years (3039), 40–49 years (4049), 50–59 years (5059), 60–69 years (6069), and ≥70 years (over70). Health conditions were divided into three categories: non-diabetes (NDM), diabetes not on dialysis (DM), and patients on dialysis due to diabetic nephropathy (DMDi). Death was represented by a flow moving outside the model (clouds). The number of individuals on dialysis due to diabetic nephropathy is considerably smaller in younger compared to older age groups (e.g., 947 males and 421 females aged ≤ 39, compared to 84,806 males and 35,470 females in total in 2015); which can reduce the stability of the model. Thus, we considered those on dialysis due to diabetic nephropathy under 40 years to be outside the model (a cloud). We prepared the model separately for both males and females.

Additional file 1: Figure S1 represents the aggregation of each stock variable (population, abbreviated as pop). In addition, NDMover20 represents individuals without diabetes aged 20 years or over; DMover20 represents individuals with diabetes not on dialysis; and DMDiover40 represents individuals with diabetes on dialysis due to diabetic nephropathy. Lastly, TotalDM represents the sum of DMover20 and DMDiover40, which estimates the number of people with diabetes (regardless of dialysis condition)

A horizontal flow represents the aging of populations within the same health condition category. Flows of NDMto20 and DMto20 are connected to stocks of NDM2029 and DM2029 respectively, indicating that those who reach adulthood enter their 20s as either non-DM or DM patients. A vertical flow represents the transfer from one health condition to another, including death (from NDM to DM, from DM to DMDi, and from all stocks to death).

### External data

Table 1 displays the data used in the simulation. We referenced Population Estimates [10] to obtain estimates of the overall population. As for sex- and age category-specific diabetes prevalence, the National Health and Nutrition Survey (NHNS) is often used as a data source [2], whereas it seems inappropriate to depend solely on the estimates of the NHNS due to several previously identified limitations [11, 12]. We therefore used estimates of sex- and age category-specific diabetes prevalence in 2000, 2005, and 2010 from a meta-regression analysis which estimated the value for each year from 2000 to 2015 [13]. We estimated sex- and age category-specific numbers of individuals without diabetes by subtracting those with diabetes from the total population. “An Overview of Regular Dialysis Treatment in Japan” (ORDTJ) of the Japanese Society for Dialysis Therapy (JSDT) was used to obtain sex- and age category-specific numbers of patients on dialysis due to diabetic nephropathy [3].

**Table 1:** The list of external data used for simulation

Explanation of variable	Unit	Data source	Period	Example of variable name in the model	Processing (if any)	Calibration or exogenous actual values
Sex- and age category- specific population estimates	Person	Population Estimates	2000–15	pop2029	Not applicable	Calibration
Sex- and age category-specific diabetes prevalence	Person	Meta-regression analysis by Charvat et al. Population Estimates	2000–15	DM2029	Estimated prevalence (%) in 2000, 2005, and 2010 were used to interpolate and extrapolate the estimates during 2000–2015. The percentages were multiplied with sex- and age category-specific population estimates.	Calibration
Sex- and age category-specific number of patients on dialysis due to diabetic nephropathy	Person	An Overview of Regular Dialysis Treatment in Japan	2000–15	DMDi4049	Sex- and age category-specific number was obtained for 2011–15. For 2000–10, the values were estimated using aggregated estimates and age- specific proportion of diabetic nephropathy among all cause in 2011–15.	Calibration
Sex-specific population estimates at the age of 20 (used as inflow toward stocks of age 20–29)	Person/year	Population Estimates	2000–35	Allto20f	The population estimate at the age of less than 20 in 2015 was used to estimate the future population at the age of 20.	Exogenous actual values
Sex-specific population estimates at the age of 40 on dialysis due to diabetic nephropathy (used as inflow toward stock of age 40–49)	Person/year	An Overview of Regular Dialysis Treatment in Japan	2000–15	DMDito40in	Sex-specific numbers in ages 35–39 and 40–44 were averaged and divided by 5.	Exogenous actual values
Sex- and age category-specific mortality rate	1/year	Vital Statistics Population Estimates An Overview of Regular Dialysis Treatment in Japan	2000–15	NDM2029drt, DM2029drt, DMDi4049drt	The mortality (incidence) was obtained from dividing the number of death by population estimates. The ratio of mortality of non-DM and DM patients were assumed to be 1:2. Actual values for patients on dialysis were used for 2012–2015 and estimated values were used for the rest of the period.	Exogenous actual values

Population Estimates were used to estimate the population at the age of 20, and ORDTJ were used to estimate the population at the age of 40 on dialysis due to diabetic nephropathy. Because a specific value for diabetes prevalence (%) at the age of 20 years was unavailable, we used 0.2% as an estimate throughout the study period from 2000 to 2035.

We collected the estimated number of deaths in the overall population from Vital Statistics [14] and in patients on dialysis due to diabetic nephropathy from ORDTJ. Research evidence suggests that the death rate among individuals with diabetes is twice as high as that among those without diabetes [15, 16]. Based on this evidence and the sex- and age category-specific prevalence of diabetes in each year, we estimated the death rate among individuals with and without diabetes. Additionally, using the previously-calculated prevalence and death rate for those on dialysis caused by diabetic nephropathy, we estimated death rates among people with diabetes but not on dialysis due to diabetic nephropathy.

For patients on dialysis, because sex- and age category-specific numbers of patients on dialysis due to diabetic nephropathy were not available for the period between 2000 and 2010, we calculated those estimates from 1) sex- and age category-specific numbers of patients on dialysis (caused by all diseases) on the year (one of the years from 2000 to 2010); 2) the average proportion of dialysis due to diabetic nephropathy among all cause between 2011 and 2015; and 3) the total number of patients on dialysis due to diabetic nephropathy on the year. For mortality of patients on dialysis, sex- and age category-specific number of death was available only for the period between 2012 and 2015; we extrapolated each trend of sex- and age category-specific mortality for the period between 2012 and 2015 to 2000–2011.

From a series of estimates obtained or generated as described above, we prepared a cross table composed of external data to be referenced during the calibration. The variables included in the cross table were as follows (all numbers were sex- and age category-specific): the population (e.g., pop2029), the number of people with diabetes but not on dialysis due to diabetic nephropathy (e.g., DM2029), and the number of people on dialysis due to diabetic nephropathy (e.g., DMDi4049). We also used the estimates from 2000 as initial values of those stock variables. The number of individuals turning age 20 per calendar year (Allto20) and the mortality rate (e.g., NDM2029dr) were also prepared and used in the model as an exogenous list of values (e.g., Allto20f, NDM2029drt). Based on the age-specific numbers of patients aged 35 to 39 years and 40 to 44 years, we estimated the number of patients on dialysis at the age of 40 years and included it as a table function (DMDito40). Since the number of individuals turning 20 is predictable through 2035 and the model is sensitive to change in this variable, we created the list of values for Allto20 for 2000 to 2035. For all other variables, lists of values were prepared for 2000 to 2015; the years following 2015 referenced the value in 2015 as a reference value.

### Calibration

The following parameters in the model were calibrated based on the external data listed above: the proportions of transfer from NDM to DM (e.g., DM2029ir, interpretable as incidence of diabetes), the proportions of transfer from DM to DMDi (e.g., DMDi4049ir, interpretable as incidence rate of dialysis initiation due to diabetic nephropathy), and the initial value of every stock variable. We investigated a sex- and age category-specific parameters applicable from 2000 to 2015; that is, we assumed that incidences of diabetes and dialysis initiation were constant throughout the study period. Calibrations were performed separately for males and females. We used the overall population (e.g., pop2029), individuals with diabetes but not on dialysis due to diabetic nephropathy (e.g., DM2029), and individuals on dialysis due to diabetic nephropathy (e.g., DMDi4049) to calculate calibration payoff. We calculated the average estimates of stock variables between 2000 and 2015 and used the inverse of these as weights in the calibration. Through calibration, we extrapolated simulation results up to 2035, which estimated the number of patients with diabetes and patients on dialysis due to diabetic nephropathy until 2035 (base run).

### Validation of the model

We examined the validity of the model in several ways. First, we checked the model fit for sex- and age category-specific stock variables in addition to aggregated stock variables. Second, we examined if the calibrated parameters were realistic by reviewing the calibrated incidence rates of diabetes and dialysis initiation due to diabetic nephropathy. When we found that some parameters were calibrated out of the expected range, we conducted sensitivity analysis on the change in these incidence rates. Third, we conducted a sensitivity analysis by changing the diabetes prevalence at the age of 20 (originally assumed as 0.2%) from 0% to 0.4% at the base run.

### Simulation experiments

**Fig. 2:**
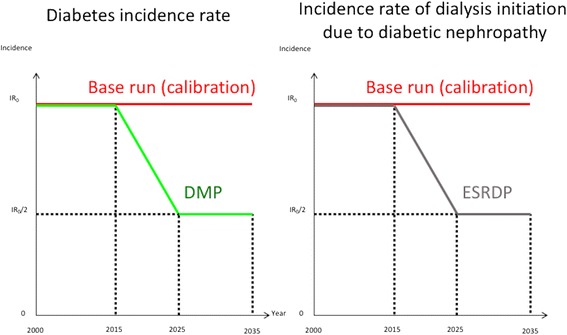
Scheme of diabetes and diabetic nephropathy incidence induced by hypothetical interventions

We performed additional simulations on three hypothetical conditions and estimated future population with diabetes and the population on dialysis due to diabetes nephropathy until 2035. First, we hypothesized that the incidence of diabetes in every sex and age category would decrease starting in 2015 due to diabetes prevention interventions (diabetes mellitus prevention; DMP) and would drop down to half of its original incidence in 2025, followed by a plateau until 2035. We assumed that diet and exercise instructions for obese people were included in the hypothetical interventions, as in the Diabetes Prevention Program that showed a beneficial effect of lifestyle intervention on the incidence of diabetes. [17] Second, we hypothesized that the incidence of dialysis initiation due to diabetic nephropathy in every sex and age category would decrease starting in 2015 due to diabetes management (end-stage renal disease prevention; ESRDP) and reach half of its original incidence by 2025. Glycemic control, blood pressure control, and protein and salt reduction among diabetes patients were assumed to be included in the hypothetical interventions. For example, intensive glucose control was shown to lower the incidence of end-stage renal disease in the ADVANCE trial [18]; moreover, the beneficial effects of blood pressure control [19, 20] and diet control [21] on renal function are also well documented. Figure 2 shows the change in sex- and age category-specific incidence during the simulation experiment. Third, we hypothesized that both interventions (DMP + ESRDP) would be conducted and that both parameters would decrease accordingly. Figure 2 shows the change in sex- and age category-specific incidence during the simulation experiment.

We performed three simulation experiments, as described above. We compared the numbers of adult patients with diabetes (TotalDM) and patients on dialysis due to diabetic nephropathy (DMDiover40) between the base run and each experimental result.

### Sensitivity analyses

We performed a couple of sensitivity analyses to confirm the robustness of the model by incorporating different model assumptions. First, an analysis extrapolating the model until 2055 was performed. Second, we conducted another analysis after incorporating exponential change in the rates of diabetes incidence and dialysis initiation.

## Results

### External data and calibration

**Fig. 3:**
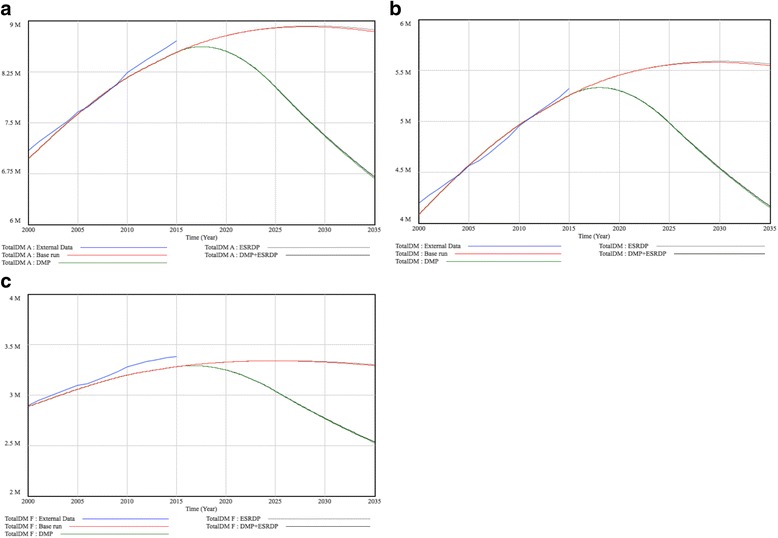
Trends of Predicted population with diabetes in Japan. **a** Total population **b** Males **c** Females

Figures 3 represents the population trends of patients with diabetes (TotalDM), both aggregately and separately for males and females. These trends include external data from 2000 to 2015 (External Data), results of calibration (base run), and the results of simulation experiments under hypothetical conditions through 2035 (DMP, ESRDP, and DMP + ESRDP). External Data showed a consistent increase in the total number of diabetes patients from 2000 to 2015 (7.09 million to 8.71 million). Sex-specific results indicated an increase during this time period from 4.19 million to 5.32 million in males, and from 2.90 million to 3.39 million in females. Calibrated to the External Data, results of calibration (base run) predicted that the population with diabetes would increase at first, but that the rate of increase would slow and would start to drop in both males and females. Specifically, the model predicted that the largest population would occur in 2028 (5.58 million males and 3.34 million females) followed by a slight drop (5.55 million males and 3.29 million females in 2035). Additional file 2: Figure S2 represents the prevalence (%) of diabetes among adults (TotalDM/popover20), which was forecasted to increase consistently until 2035.

**Fig. 4:**
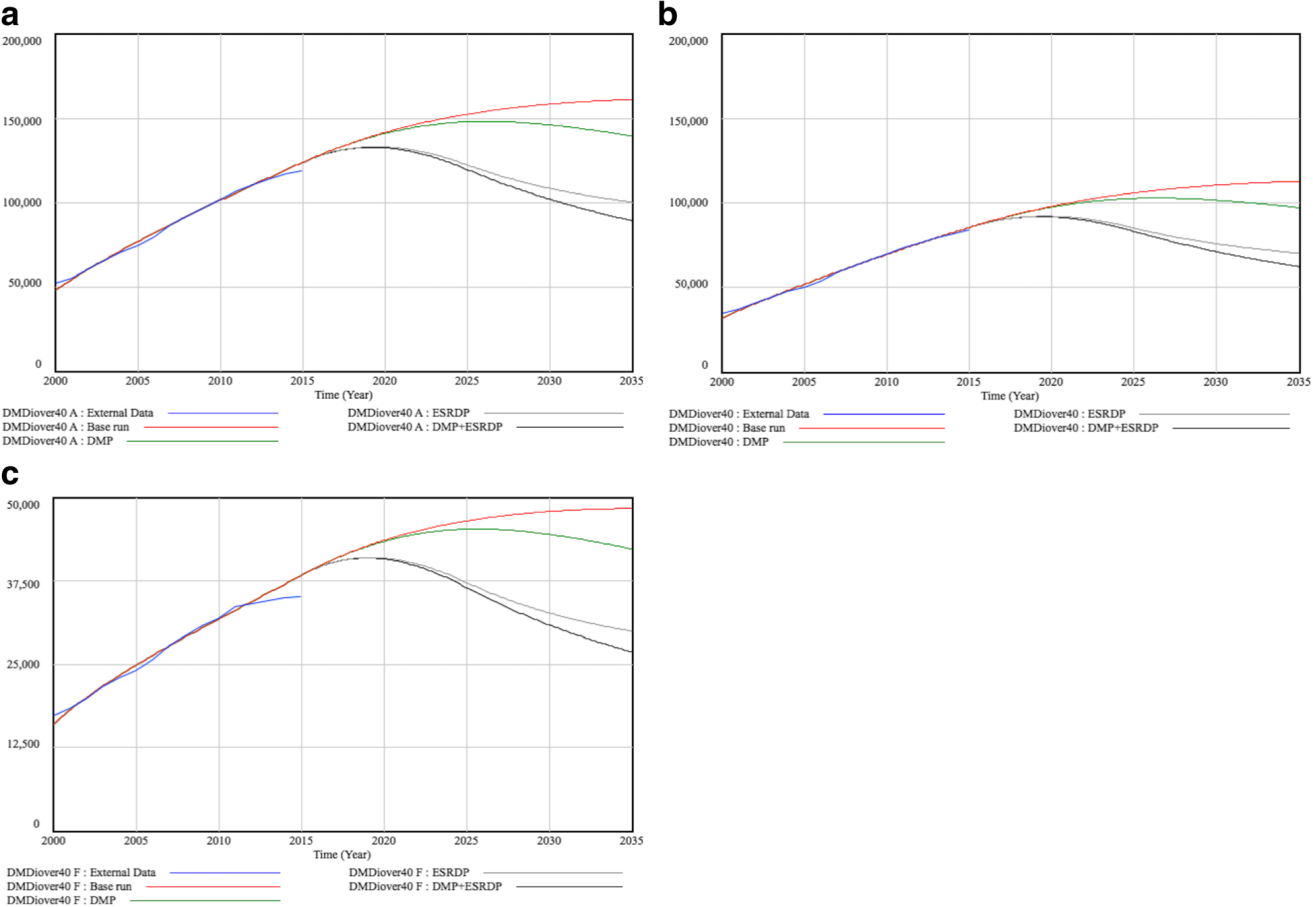
Trends of predicted population on dialysis due to diabetic nephropathy in Japan**. a** Total population **b** Males **c** Females

Figures 4 represents the population trends of patients on dialysis due to diabetic nephropathy (DMDiover40), both aggregately and separately for males and females. External Data showed an increase in the total number of patients on dialysis due to diabetic nephropathy between 2000 and 2015 (51,891 to 118,906). Sex-specific data indicated an increase during this time period from 34,672 to 83,858 in males, and from 17,219 to 35,048 in females. The result of calibration (base run) forecasted that the population on dialysis due to diabetic nephropathy would increase consistently. The model predicted that 112,685 males and 48,389 females would be living on dialysis due to diabetic nephropathy in 2035.

### Validation of the model

As for the model fit for sex- and age category-specific stock variables, we found that some of the variables did not fit the external data very well, especially in the young age group. On the other hand, the model fit for the aggregated population was very good and should be useful for comparing the simulated trends.

Next, we examined if the calibrated parameters were realistic. As shown in the Additional file 3: Table S1, the incidence rates of diabetes and dialysis initiation increase by age in most categories, and it is reasonable that for the most part, the incidence of diabetes and that of dialysis among males was higher than that among females. The only exceptions were the incidence rate of diabetes (DM3039ir) among females aged 30–39 and the incidence rate of dialysis initiation due to diabetic nephropathy among females aged 40–49 (DMDi4049ir), which were calibrated as 0. We therefore conducted additional sensitivity analyses; we incorporated changes in these parameters from 0 up to the level of next age group (0.00138225 for DM3039ir and 0.00136167 for DMDi4049ir). The Additional file 4: Figure S3 and Additional file 5: Figure S4 present the results of the sensitivity analyses. As shown above, the changes in these rates (within a realistic range) did not have a major influence on the trends. Based on these results, we decided not to change the rates from the originally calibrated values.

We also conducted a sensitivity analysis to test the robustness of the model using different prevalence rates of diabetes at the age of 20 (Additional file 6: Figure S5). We found that the prevalence of diabetes at all ages (20 years or older) increased on using a higher prevalence of diabetes at the age of 20 (i.e., 0.4% rather than 0.2% or 0%); however, the resultant difference in the estimated population with diabetes in 2035 was quite small (8.796 million at 0% prevalence; 8.828 million at 0.2% prevalence; and 8.868 million at 0.4% prevalence).

### Simulation experiments

Figures 3 and 4 display the results of simulation experiments, in which parameters were replaced on the assumption that hypothetical interventions would decrease the incidence of diabetes and/or dialysis initiation due to diabetic nephropathy. When we assumed that diabetes prevention interventions (DMP) only were performed successfully by 2025 and that the decreased incidence would remain stable until 2035, it was forecasted that the numbers of diabetes patients would begin to decline within a few years. In 2035, the numbers reached 4.15 million in males and 2.53 million in females (reductions of 25.2% and 23.3% in males and females, respectively from base run results). However, DMP alone would not have a great impact on the number of patients on dialysis due to diabetic nephropathy; the projected lines of DMP and the base run would almost overlap until approximately 2025. After 2025, it was projected that the two lines would begin to diverge, dropping to 97,138 in males and 42,281 in females (13.8% and 12.6% reductions, respectively). For interventions to decrease dialysis initiation (ESRDP), we suspected that the number of diabetes patients would not decline at all (5.57 million males and 3.30 million females in 2035); whereas, the numbers of dialysis initiations due to diabetic nephropathy would decrease considerably (70,181 males; a 37.7% decrease, and 29,931 females; a 38.1% decrease in 2035). When we combined both interventions (DMP + ESRDP), the number of diabetes patients was projected to decrease at almost the same rate as the significant declines found in DMP, whereas the number of patients on dialysis due to diabetic nephropathy was projected to decrease at the fastest rate among the 4 simulation cases.

### Sensitivity analyses

We performed another analysis extrapolating the model until 2055. We found that the difference in predicted total population with diabetes between the groups with and without DMP would become wider after 2035 (Additional file 7: Figure S6a). In contrast, the difference in predicted total population with dialysis due to diabetic nephropathy between the group with DMP (without ESRDP) and the group with ESRDP (without DMP) would become smaller after 2035 (Additional file 7: Figure S6b).

The other sensitivity analysis which incorporated exponential changes in diabetes incidence rates and dialysis initiation rates showed that future population with diabetes and population on dialysis decreased due to the relaxed condition. However, the inter-relationship among the four situations did not change (Additional file 8: Figure S7).

## Discussion

In this study, we constructed an aging chain simulation model of diabetes incidence and complication progression, focusing on dialysis initiation due to diabetic nephropathy using system dynamics method. We estimated that the total number of diabetes patients in 2022 would be 8.8 million, followed by a slight increase to 8.9 million in 2028 and a slight decrease to 8.8 million in 2035. In the government-initiated health-promotion campaign called Health Japan 21 [22], the Ministry of Health, Labour and Welfare predicted that in the absence of any intervention, the number of adults strongly suspected to have diabetes would increase up to 14.1 million by 2022. The prediction was based on extrapolation of regression lines of sex- and age category-specific prevalence rates in logit transformation. The target goal, which was based on NHNS data by 2007 and was announced in Health Japan 21, was 10 million diabetes patients in 2022 [23]. Based on our predictions, this goal could have been achieved without any intervention. However, the predictions and goals of Health Japan 21 were determined based solely on NHNS estimates (i.e., 7.4million in 2002 and 8.9 million in 2007); whereas, the article by Charvat et al. [13] estimated a prevalence of 8.3 million in 2010. It is unsurprising that the estimates in our base run based on the article by Charvat et al. [13] were quite lower than the estimates in Health Japan 21 goal determined based on the NHNS.

An important finding of our study was that from a short-term perspective (20 years), diabetes prevention interventions were rather ineffective at reducing the number of patients on dialysis due to diabetic nephropathy. This type of intervention would become effective in the long run via a reduction in diabetes prevalence. Jones et al. [7] made predictions based on data up to 2004 to portray futures through 2050, which demonstrated the greater effectiveness of diabetes prevention via obesity prevention than increased management of prediabetes or enhanced clinical management of diabetes. In the sensitivity analysis extrapolating the model until 2055, we found that the effect of DMP on dialysis prevalence would become larger decades later. In order to understand the impact of diabetes prevention intervention correctly, a very long-term observation or simulation would be required.

We also found that the hypothetical ESRDP quite evidently stopped the rapidly increasing prevalence of patients on dialysis due to diabetic nephropathy. The hypothetical intervention decreased the population on dialysis by 38% compared to the base run. However, it is predicted that this effect will not be obvious for the first decade, suggesting that the economic burden of dialysis will last for at least another one to two decades.

The validation of the model showed that the calibrated model was generally reasonable and valid. Also, the sensitivity analyses showed that our model was quite robust against the different assumption about the prevalence of diabetes at the age of 20.

In this study, we used system dynamics model for model building and prediction of future population. System dynamics modeling is a particularly powerful tool when the focus of analysis is on the aggregate characteristics of the population. For projection of patients with diabetes, Wong et al. projected diabetes population in Singapore using a dynamic Markov model. [22] Their Markov model requires individual transition probability between states, and most of these were forecasted based on additional assumptions. Our system dynamics model incorporated fewer assumptions and the rates of transition (flow) were estimated by calibration. In Health Japan 21, the Japanese government predicted future population with diabetes in Japan by extrapolating regression lines of sex- and age category-specific prevalence rates in logit transformation; [23] in contrast, our model allowed incorporation of non-linear change and also allowed for simulation experiments by manipulating incidence rates.

Our study has several limitations. First, the results are based on many assumptions. For example, in the base run, we did not allow the diabetes incidence rate and dialysis initiation rate to change within the study period; this assumption would not be realistic. Given the population’s increasing consciousness towards maintaining a healthy lifestyle and thanks to medical progress, it is natural to assume that dialysis rates have gradually decreased and will continue to decline in the foreseeable future. For this assumption, we conducted a sensitivity analysis after incorporating exponential change in the incidence rates of diabetes and dialysis initiation. The resultant model showed slightly-decaying incidence rates of diabetes and dialysis, whereas the main conclusion of this study was robust against this type of change. Secondly, we did not focus on those who initiated dialysis due to conditions other than diabetic nephropathy. Individuals may receive dialysis to treat other conditions such as chronic glomerulonephritis without diabetes, and those who have diabetes may begin dialysis as a result of other kidney diseases. Third, we did not include those on dialysis due to diabetic nephropathy under 40 years. This is because inclusion of the stock variables with small numbers would lead to unreasonably large weight on the variables that would make the model less stable.

## Conclusions

We predicted the number of people with diabetes and the number of people on dialysis due to diabetic nephropathy up to 2035 based on an aging chain simulation model. The model suggests that the prevalence of diabetes would plateau around late 2020s, while the increase in the number of people on dialysis due to diabetic nephropathy would last during the subsequent 20 years. Further simulation experiments using the model suggested that when examining the change 20 years after the start of an effective intervention, a diabetes prevention intervention would have a smaller impact on the number of dialysis initiations than direct dialysis prevention intervention. Interventions aiming at prevention of dialysis initiation would have a larger and faster impact on the number of dialysis initiations, at least within the scope of our prediction. Our model implied that it would take more than 20 years for an effective diabetes prevention intervention to decrease the number of patients receiving dialysis due to diabetic nephropathy via a reduction in the number of diabetes patients. Policymakers will need to have a long-term perspective when they consider future interventions with regard to diabetes.

## Additional files

Additional file 1: Figure S1. Aggregation of variables in the model. (TIFF 415 kb)

Additional file 2: Figure S2. Trends of prevalence of diabetes in Japan (%). a) Total. b) Males. c) Females. (TIFF 28569 kb)

Additional file 3: Table S1. Calibrated incidence rates of diabetes and dialysis initiation due to diabetic nephropathy. (DOCX 11 kb)

Additional file 4: Figure S3. Sensitivity analysis incorporating the change in incidence rate of diabetes among females aged 30–39 – from 0 to the value among females aged 40–49. Total population with diabetes. (TIFF 288 kb)

Additional file 5: Figure S4. Sensitivity analysis incorporating the change in incidence rate of dialysis initiation among females aged 40–49 – from 0 to the value among females aged 50–59. Total population with dialysis due to diabetic nephropathy (TIFF 349 kb)

Additional file 6: Figure S5. Trends of predicted population with diabetes in Japan – sensitivity analysis after incorporation of changes in the prevalence of diabetes at the age of 20. (TIFF 450 kb)

Additional file 7: Figure S6. Extrapolation of the model up to 2055. a) Trends of predicted population with diabetes. b) Trends of predicted population on dialysis due to diabetic nephropathy. (TIFF 28569 kb)

Additional file 8: Figure S7. Sensitivity analysis after incorporating changes in diabetes incidence rates and dialysis initiation rates – from constant to allowing exponential change. a) Total population with diabetes. b) Total population on dialysis due to diabetic nephropathy. (TIFF 28569 kb)

## Abbreviations

DM: Diabetes not on dialysis
DMDi: Patients on dialysis due to diabetic nephropathy
DMP: Diabetes mellitus prevention
ESRDP: End-stage renal disease prevention
IDF: International Diabetes Federation
JSDT: The Japanese Society for Dialysis Therapy
NDM: Non-diabetes
NHNS: National Health and Nutrition Survey
ORDTJ: Overview of Regular Dialysis Treatment in Japan
