# Supplementary figures and images for: Construction of a simulation model and evaluation of the effect of potential interventions on the incidence of diabetes and initiation of dialysis due to diabetic nephropathy in Japan
Source: BMC Health Serv Res. 2017 Dec 16;17:833. doi: 10.1186/s12913-017-2784-0 (PMC5732509; doi:10.1186/s12913-017-2784-0)

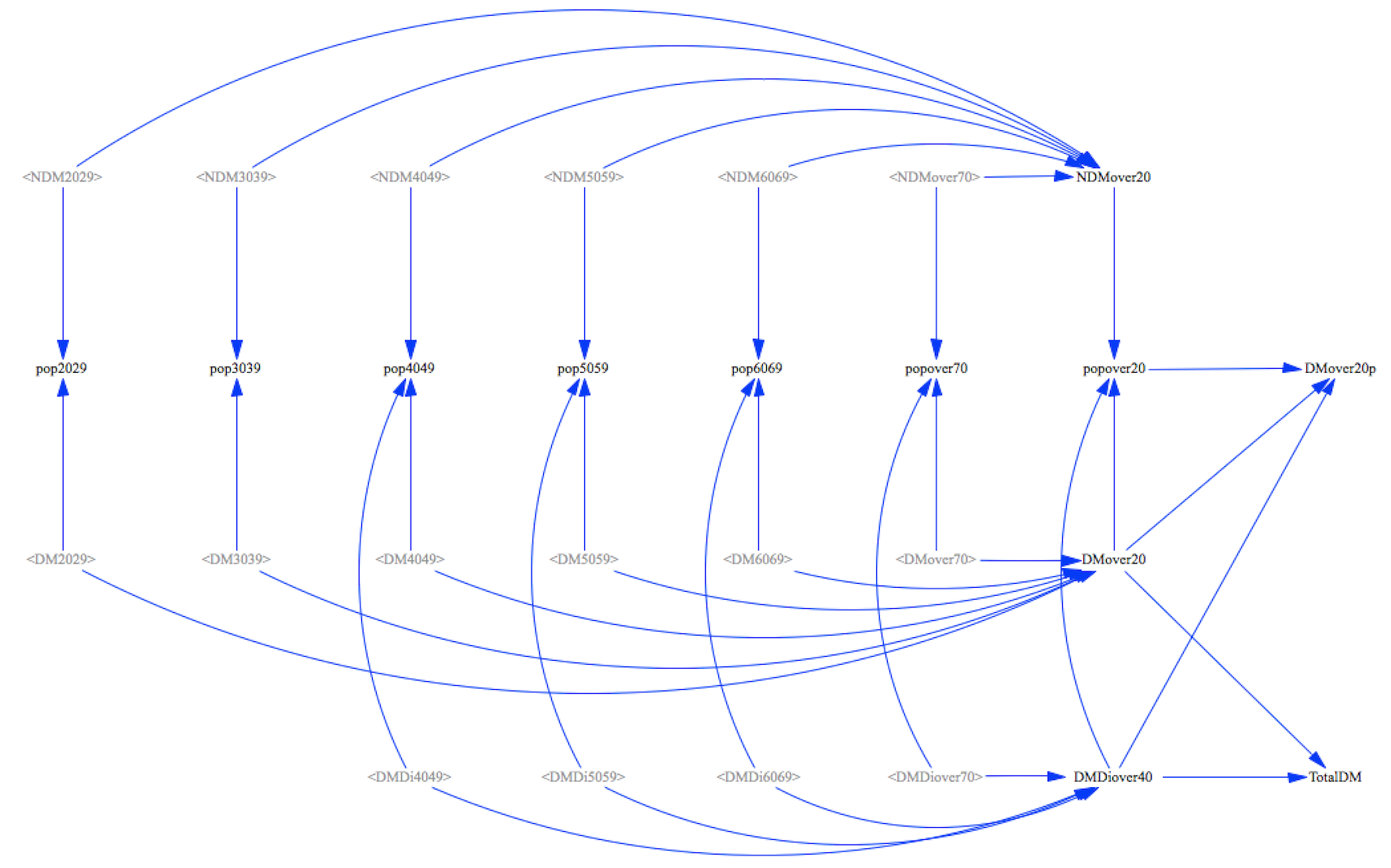

Supplement: Supplementary file 1 — Aggregation of variables in the model. (TIFF 415 kb) [file 12913_2017_2784_MOESM1_ESM.tif]

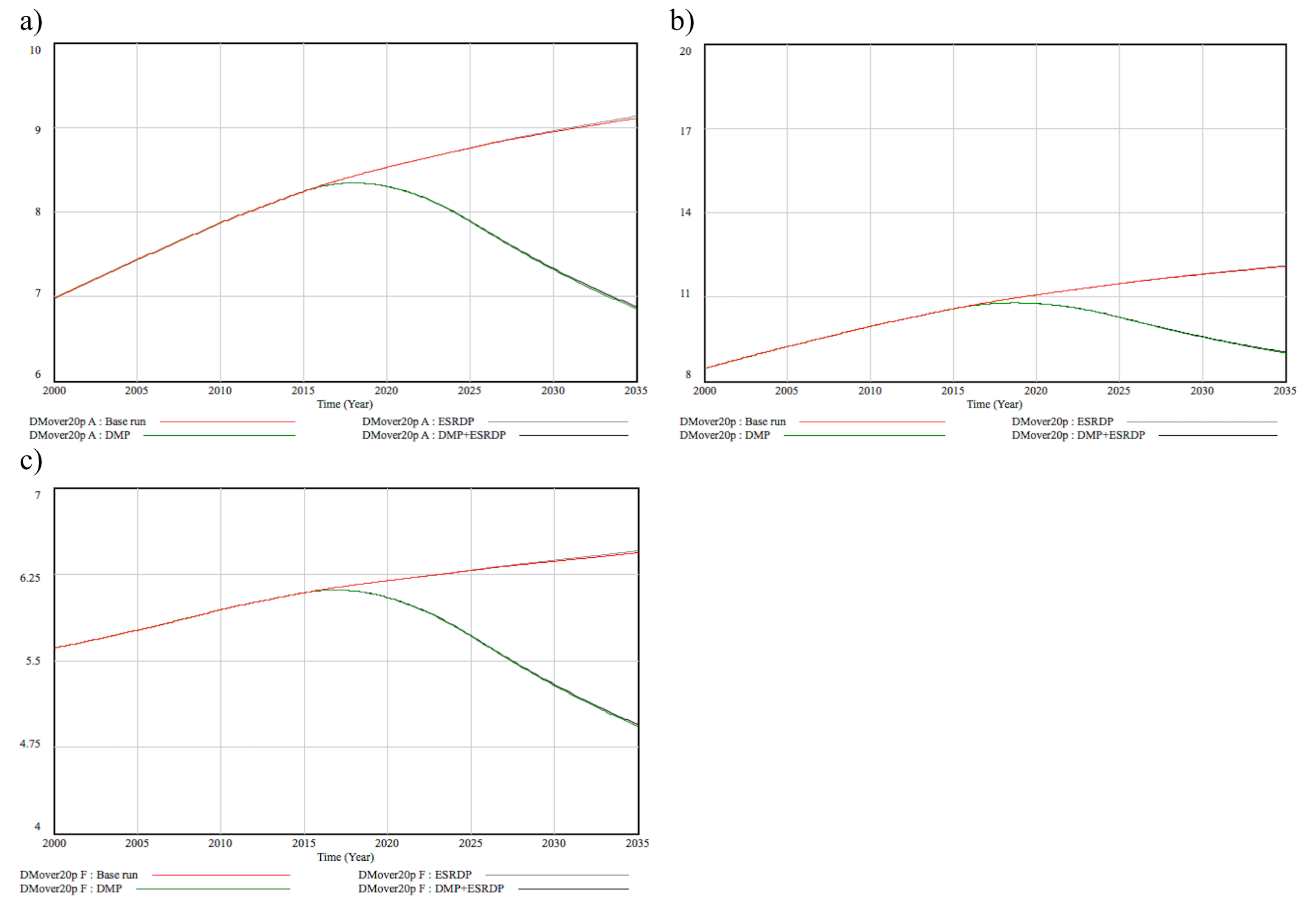

Supplement: Supplementary file 2 — Trends of prevalence of diabetes in Japan (%). a) Total. b) Males. c) Females. (TIFF 28569 kb) [file 12913_2017_2784_MOESM2_ESM.tiff]

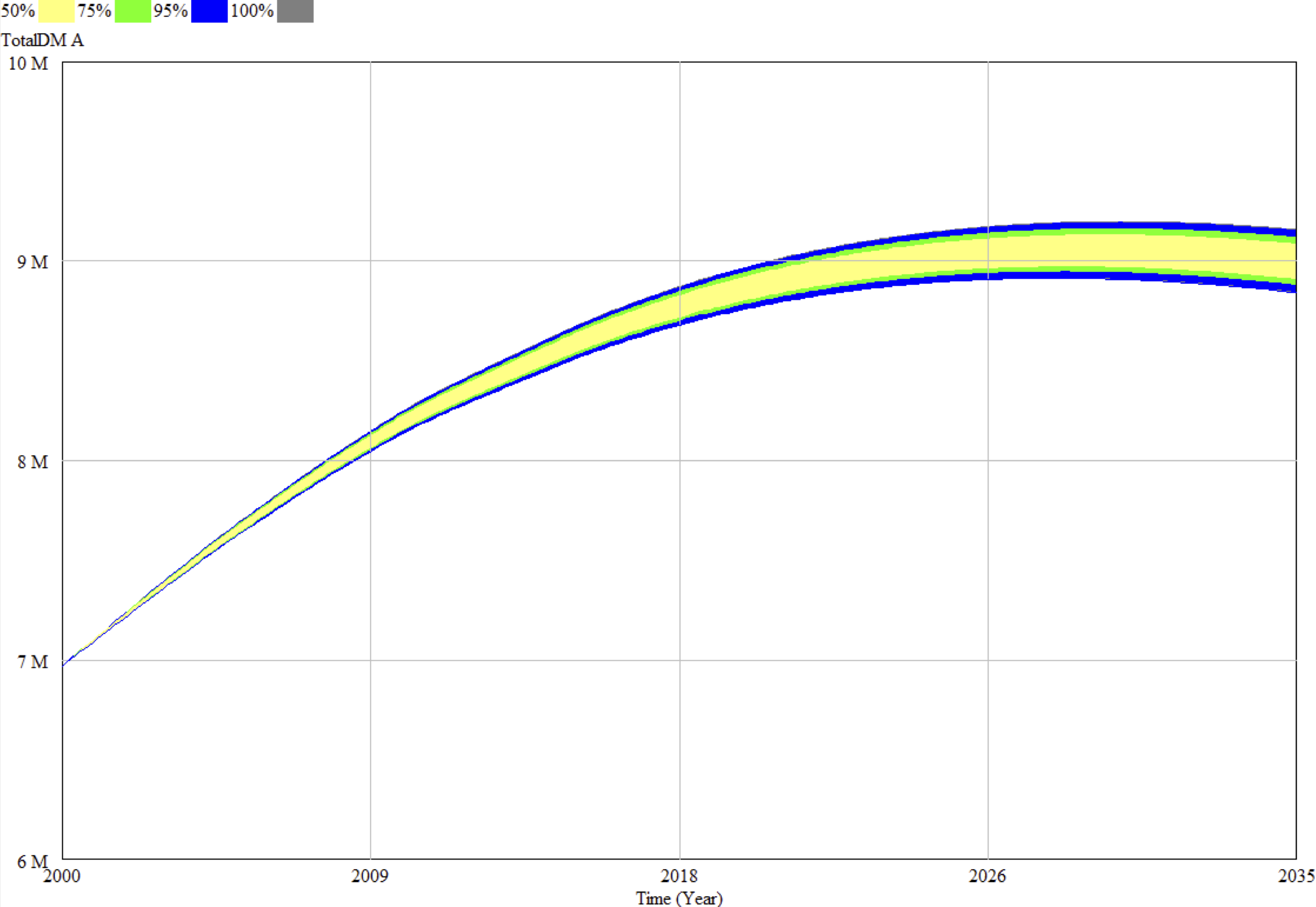

Supplement: Supplementary file 4 — Sensitivity analysis incorporating the change in incidence rate of diabetes among females aged 30–39 – from 0 to the value among females aged 40–49. Total population with diabetes. (TIFF 288 kb) [file 12913_2017_2784_MOESM4_ESM.tiff]

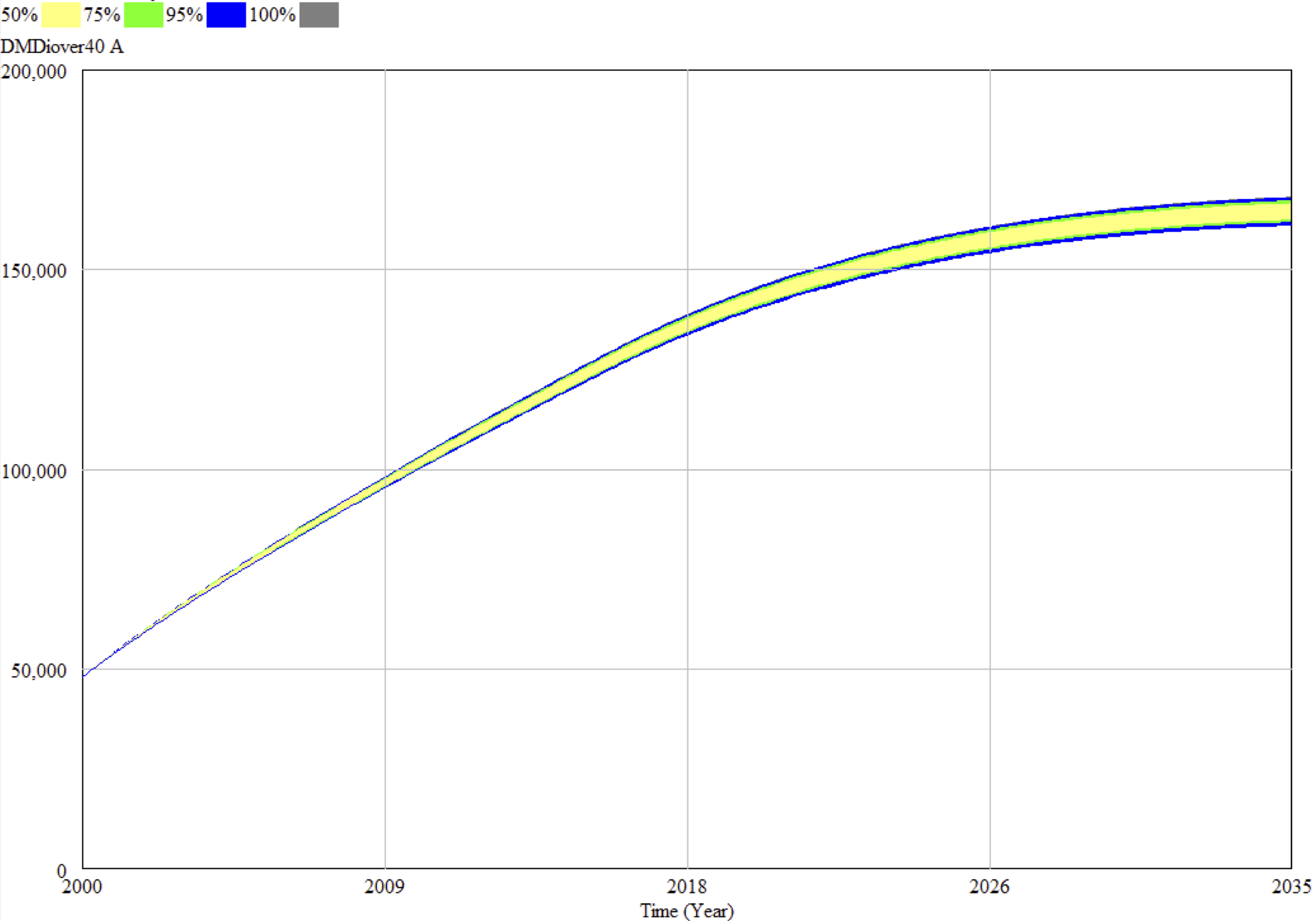

Supplement: Supplementary file 5 — Sensitivity analysis incorporating the change in incidence rate of dialysis initiation among females aged 40–49 – from 0 to the value among females aged 50–59. Total population with dialysis due to diabetic nephropathy (TIFF 349 kb) [file 12913_2017_2784_MOESM5_ESM.tiff]

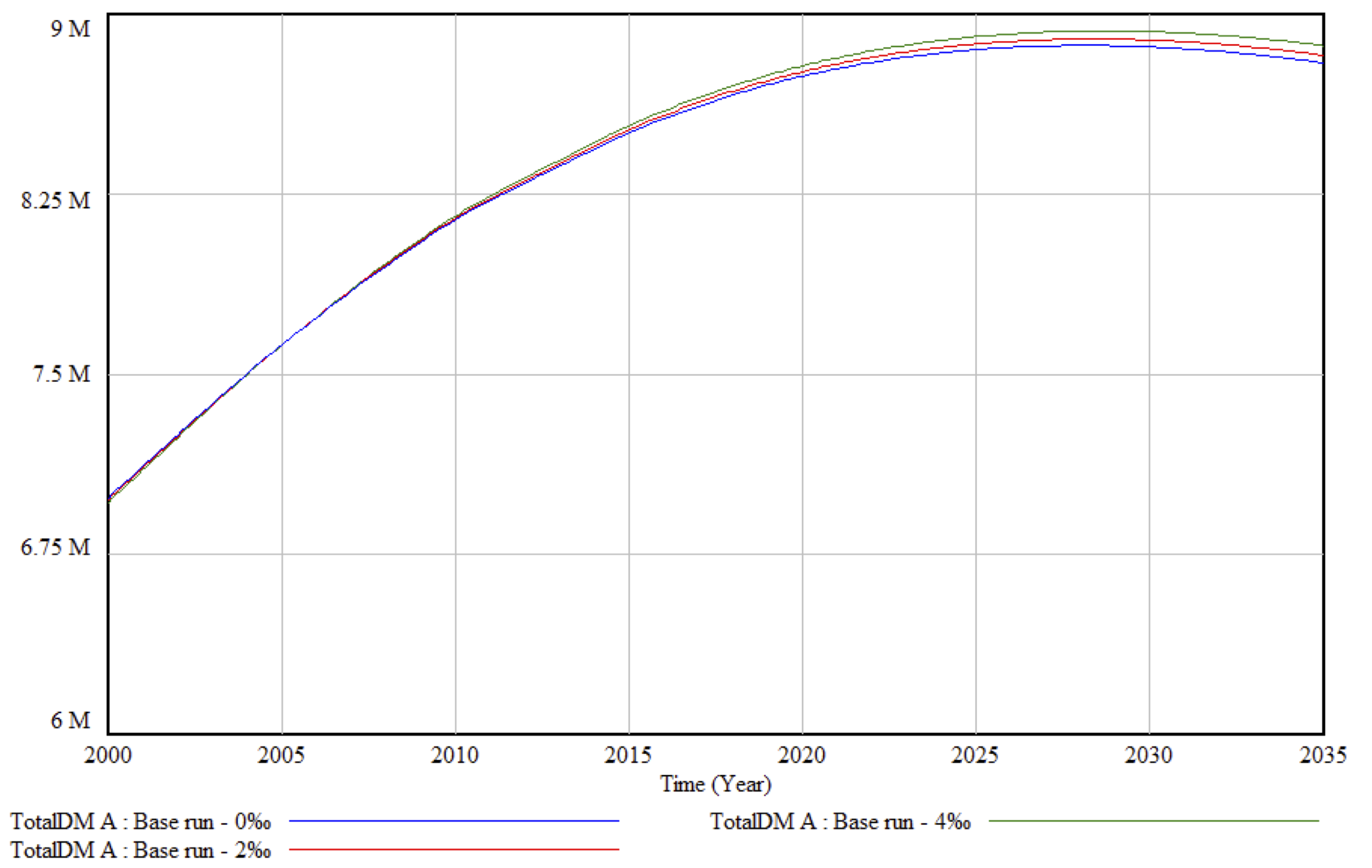

Supplement: Supplementary file 6 — Trends of predicted population with diabetes in Japan – sensitivity analysis after incorporation of changes in the prevalence of diabetes at the age of 20. (TIFF 450 kb) [file 12913_2017_2784_MOESM6_ESM.tiff]

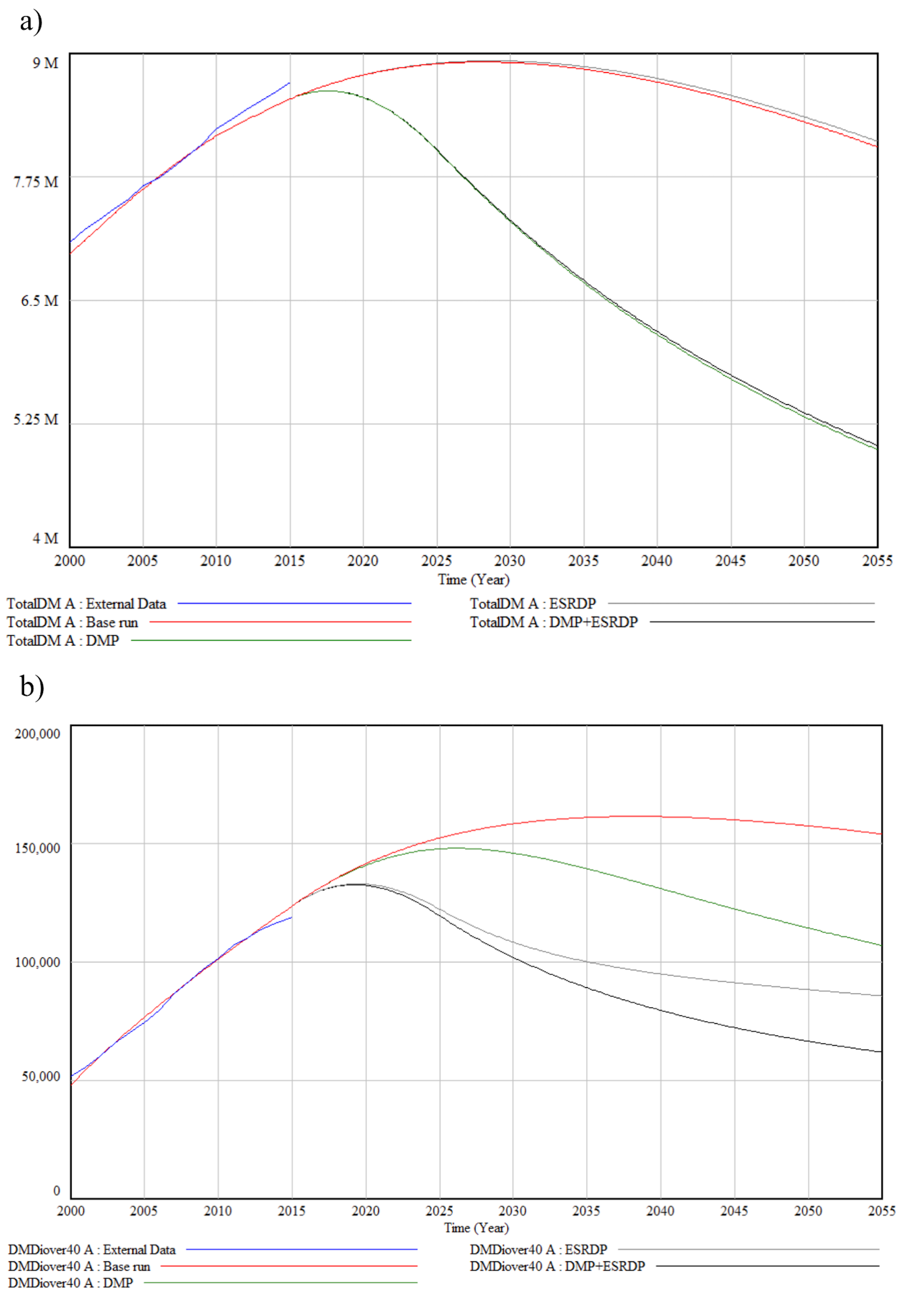

Supplement: Supplementary file 7 — Extrapolation of the model up to 2055. a) Trends of predicted population with diabetes. b) Trends of predicted population on dialysis due to diabetic nephropathy. (TIFF 28569 kb) [file 12913_2017_2784_MOESM7_ESM.tiff]

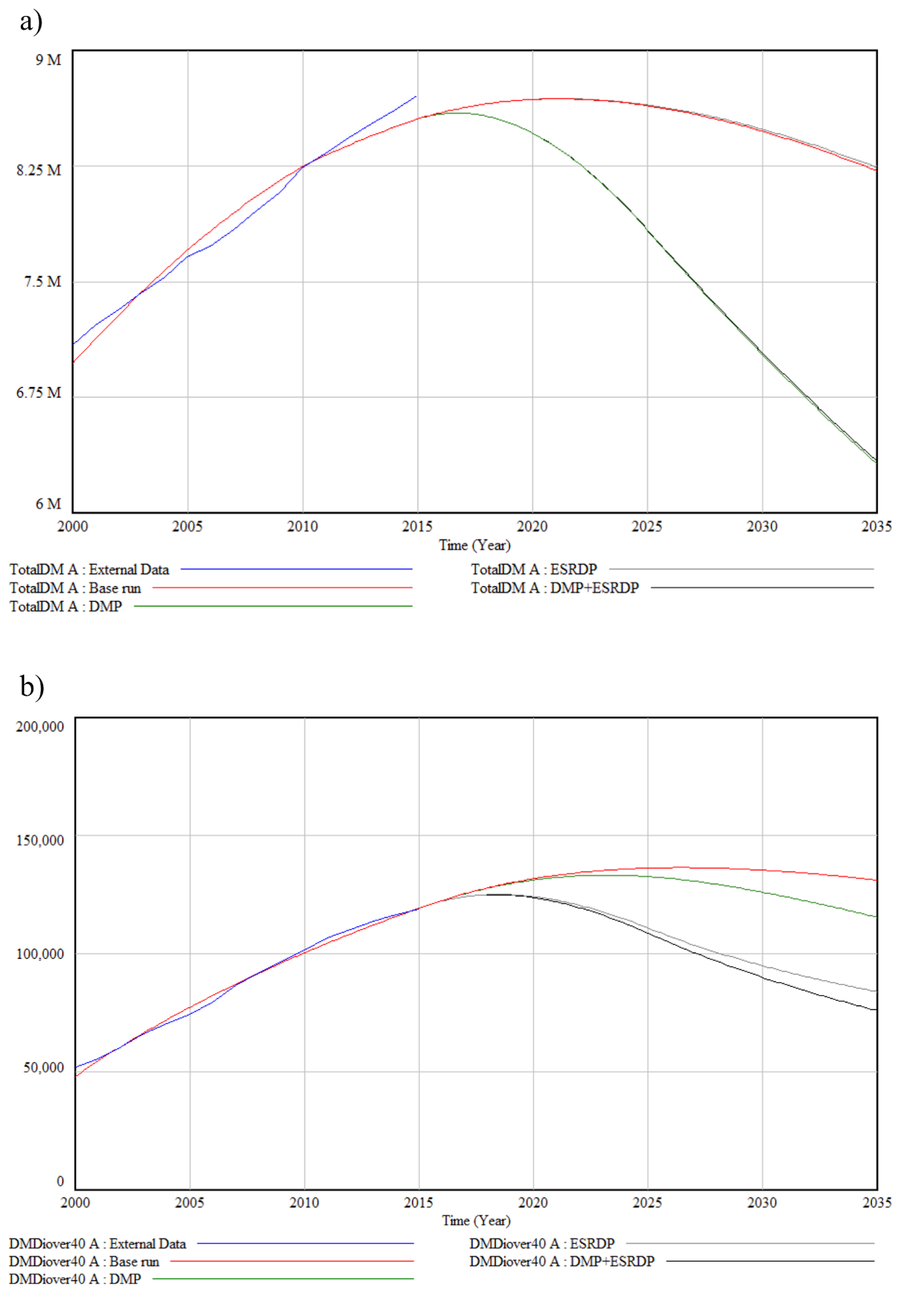

Supplement: Supplementary file 8 — Sensitivity analysis after incorporating changes in diabetes incidence rates and dialysis initiation rates – from constant to allowing exponential change. a) Total population with diabetes. b) Total population on dialysis due to diabetic nephropathy. (TIFF 28569 kb) [file 12913_2017_2784_MOESM8_ESM.tiff]
