# Supplementary material for: Construction of a simulation model and evaluation of the effect of potential interventions on the incidence of diabetes and initiation of dialysis due to diabetic nephropathy in Japan
Source: BMC Health Serv Res. 2017 Dec 16;17:833. doi: 10.1186/s12913-017-2784-0 (PMC5732509; doi:10.1186/s12913-017-2784-0)
Supplement: Supplementary file 3 — Calibrated incidence rates of diabetes and dialysis initiation due to diabetic nephropathy. (DOCX 11 kb) [file 12913_2017_2784_MOESM3_ESM.docx]

Additional Table 1 Calibrated incidence rates of diabetes and dialysis initiation due to diabetic nephropathy.

|  | Incidence rate of diabetes | | Incidence rate of dialysis initiation | |
| --- | --- | --- | --- | --- |
| Age category | Male | Female | Male | Female |
| 20-29 | 0.000265305 | 0.000265305 | - | - |
| 30-39 | 0.00073863 | 0 | - | - |
| 40-49 | 0.00351798 | 0.00138225 | 0.0006292986 | 0 |
| 50-59 | 0.0055264 | 0.00144019 | 0.00163167 | 0.00136167 |
| 60-69 | 0.0176086 | 0.0091531 | 0.00242874 | 0.00153566 |
| Over 70 | 0.0100526 | 0.00340989 | 0.00260032 | 0.00191494 |
